# Supplementary material for: CasTuner is a degron and CRISPR/Cas-based toolkit for analog tuning of endogenous gene expression
Source: Nat Commun. 2023 Jun 3;14:3225. doi: 10.1038/s41467-023-38909-4 (PMC10239436; doi:10.1038/s41467-023-38909-4)
Supplement: Supplementary file 1 — Supplementary Information [file 41467_2023_38909_MOESM1_ESM.pdf]

CasTuner is a degron and CRISPR/Cas-based toolkit for analog tuning of endogenous gene expression

Supplementary Information

Gemma Noviello, Rutger AF Gjaltema, Edda G Schulz

Supplementary Figure 1

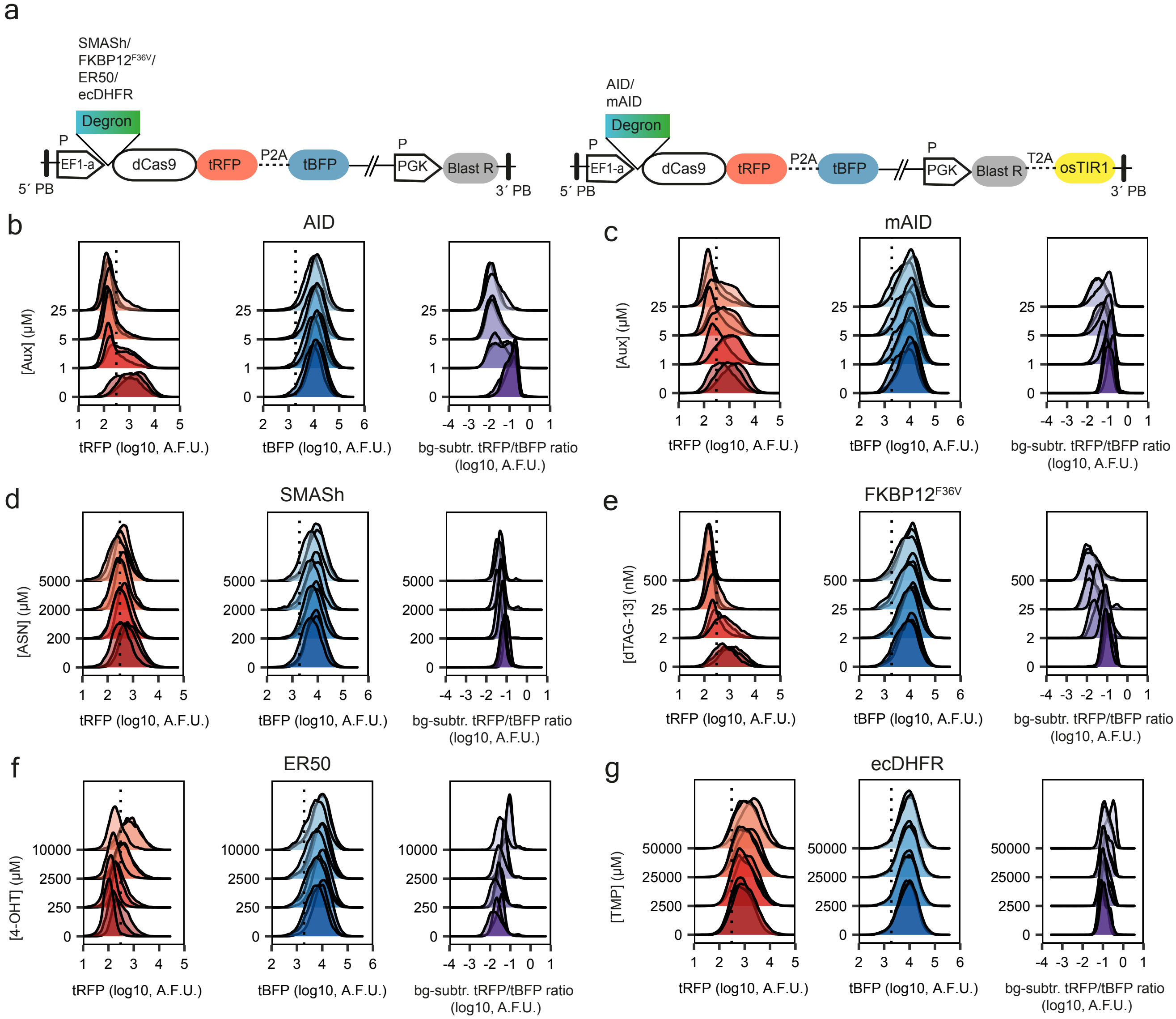

Supplementary Figure 1: Comparison of degron domains to control dCas9.

a, A detailed schematic of degron-dCas9-tRFP-P2A-tBFP plasmids. The region between the 5’ and 3’ PB sites is genomically integrated. The degrons SMASh, FKBP12^{F36V}, ER50 and ecDHFR (left) do not require any accessory protein for their degradation. The blasticidin resistance gene is expressed under a separate promoter (PGK). The no-degron control plasmid is based on the same design. The AID and mAID degrons (right) require the TIR1 protein from Oryza sativa (OsTIR1), which is expressed under the same promoter of the blasticidin resistance, separated by a T2A site.

b-g, Density plots showing the fluorescence distributions across cells for tRFP, tBFP and the tRFP/tBFP ratio at different ligand concentrations for each degron domain as indicated. Three biological replicates for each concentration are overlaid. A.F.U. Arbitrary Fluorescence Units.

Supplementary Figure 2

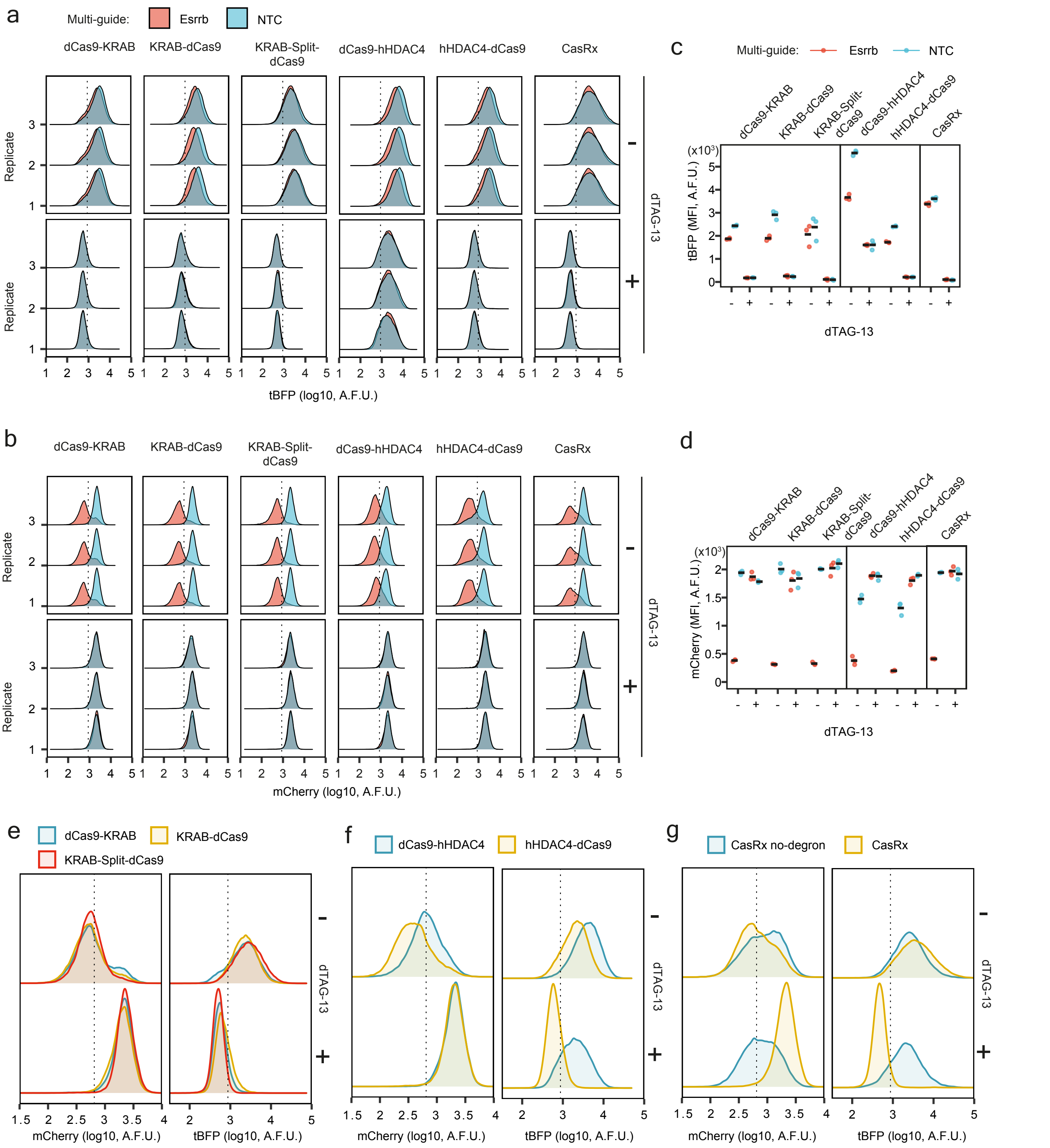

Supplementary Figure 2: Testing inducibility and efficiency of repression through degron-Cas-repressors in an endogenous reporter system

a,**b**, Density plots of tBFP (**a**) or mCherry levels (**b**) in cells containing Esrrb- (red) or non- (blue) targeting guides, in -dTAG-13 (top) or +dTAG-13 (bottom) conditions, measured by flow cytometry.

c,**d**, Background-subtracted MFI of tBFP (**c**) or mCherry (**d**) for degron-Cas-repressor cell lines indicated on top, in - or +dTAG-13 conditions. For Esrrb- (red) or non- (blue) targeting guides. Dots showing 3 biological replicates and horizontal bars their mean.

e-**g**, Density plots showing the overlaid distributions for mCherry (left) and tBFP (right), in -dTAG-13 (top) and +dTAG-13 condition (bottom) in cells expressing Esrrb-targeting guides for the three KRAB-mediated repression systems (**e**) the two hHDAC4-based systems (**f**) and the CasRx system (**g**). For the degron-CasRx construct, the same construct lacking the degron domain (CasRx no-degron) was included for comparison. The three biological replicates are merged together.

In **a**,**b**,**e**-**g** the dotted line indicates the 99th percentile of non-fluorescent control cell line. A.F.U.= arbitrary fluorescence units.

Supplementary figure 3

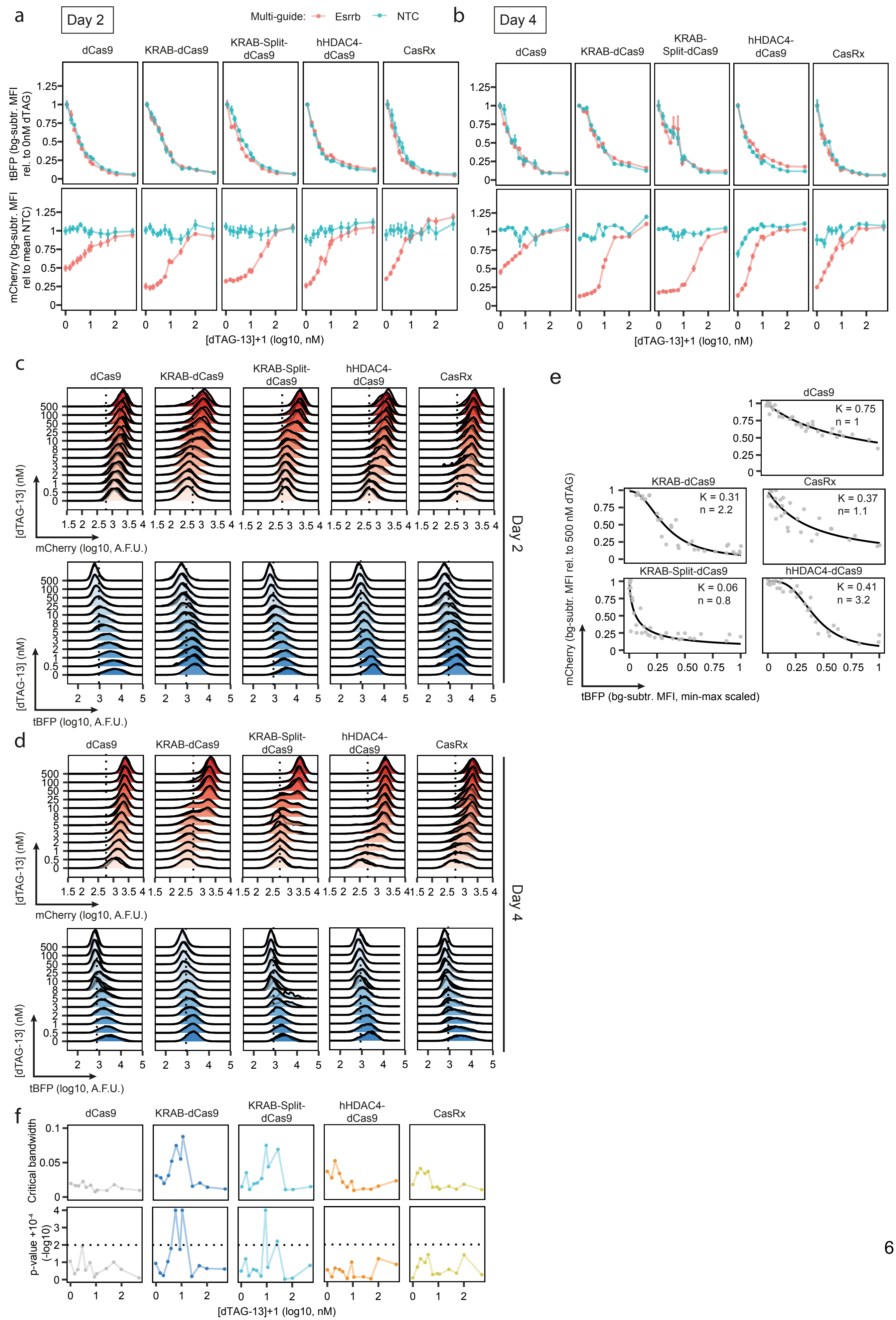

Supplementary Figure 3: Assessing tunability of degron-Cas-repressor systems

a, **b**, tBFP (top) and mCherry (bottom) fold change of background subtracted MFI in cells expressing Esrrb-targeting guides (red) and cells expressing NTC guides, at different dTAG-13 concentrations (y-axis, log10 +1 scaled, in nM) after 2 (**a**) or 4 (**b**) days of treatment. The fold change is calculated relative to the mean of the MFI in cells with non-targeting guides at the different dTAG-13 concentrations. The mean of three biological replicates (dots) ± standard deviation (vertical bars) is shown. Lines connect the means.

d, **c**, Density plots of mCherry (top) or tBFP (bottom) expression levels measured by flow cytometry at increasing dTAG-13 concentrations (y-axis, in nM) for degron-Cas-repressors indicated on top in cells expressing Esrrb-targeting guides at 2 (**c**) and 4 (**d**) days of treatment. Three biological replicates are overlaid. dCas9 and KRAB-Split-dCas9 are the same construct (KRAB-Split-dCas9) without or with addition of 100µM ABA, respectively.

e, Hill function fitted to titration experiments at the 4 day time point. Shown is the mCherry fold change (y-axis) at varying tBFP levels (min.-max. scaled, x-axis) for all replicates and dTAG-13 concentrations (grey dots) and the best fitting Hill-type equation (black line) resulting from a non-linear least square (NLS) approach to find the parameters K (repression coefficient) and n (Hill coefficient), which are reported inside each plot.

f, Test for unimodality of the mCherry distributions shown in (**d**). Critical bandwidth (top) and associated p-value (bottom) at different dTAG-13 concentrations, calculated with the test for multimodality proposed by Hall and York (see Methods). Null hypothesis is that distributions are unimodal. The horizontal dotted line indicates a p-value of 0.01.

Supplementary figure 4

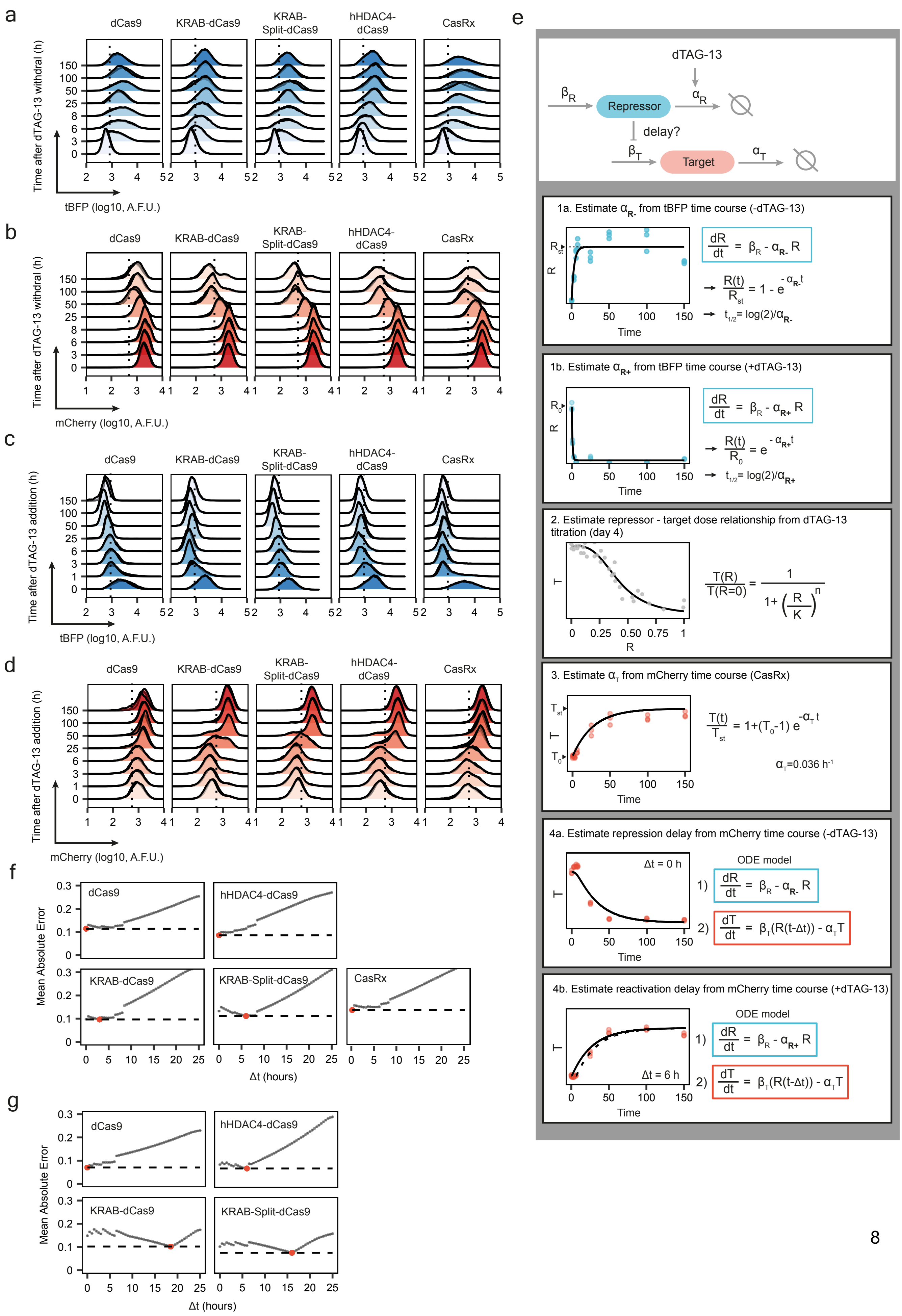

Supplementary Figure 4: Measuring the dynamics of repression and reversibility associated with degron-Cas-repressor systems

a, **b**, tBFP (**a**) and mCherry (**b**) levels measured by flow cytometry (x axis) at different time points (y axis, in hours from dTAG-13 withdrawal) for the experiment performed to measure the dynamics of repression. Different repressor systems are indicated on top. Three biological replicates are overlaid.

c, **d** tBFP (**c**) and mCherry (**d**) levels measured by flow cytometry (x axis) at different time points (y axis, in hours from dTAG13 addition) for the experiment performed to measure the dynamics of repression reversibility. Different repressor systems are indicated on top. Three biological replicates are overlaid.

e, Modelling approach used to quantify the dynamic of target gene repression and derepression for the different degron-Cas-repressors. Repressor (R) and Target gene (T) dynamics were simulated with an ODE model, describing production with rate □ and degradation with rate α for both R and T. In step 1, repressor degradation rates in the presence (α_{R+}) and absence (α_{R-}) of dTAG-13 and the resulting half life ($t_{1/2}$) were estimated by fitting the tBFP time course in the presence (1b) or absence (1a) of dTAG-13 with the equation describing the repressor as indicated. In step 2, the relationship between target gene expression and repressor level at the steady state is modelled by fitting an Hill curve to the titration data from Supplementary Fig. 3e. In step 3, the mCherry derepression time course of the CasRx cell line is used to estimate the mCherry degradation rate (α_T). All the parameters estimated in steps 1-3 are then combined in step 4a and 4b to simulate a model of two ODEs to describe the dynamic of repression and derepression, respectively. By comparing experimental data with simulations, assuming different delays (Δt) between repressor up- or downregulation and effects on target gene expression, the value for Δt that could best reproduce the data was identified.

f, Mean absolute error (MAE) for the simulations of the ODE model used for studying the repression dynamic of different degron-Cas-repressors, for different Δt. Each grey dot represents the MAE of the simulation for the corresponding Δt. The Δt associated with the lowest MAE is marked with a red dot and the dashed line indicates the corresponding MAE.

g, As in (**f**), but for the models used to simulate the reversibility dynamics. Here the MAE is not shown for CasRx because the CasRx experiment was used to calculate the degradation rate of the target protein ESRRB-mCherry.

Supplementary Figure 5

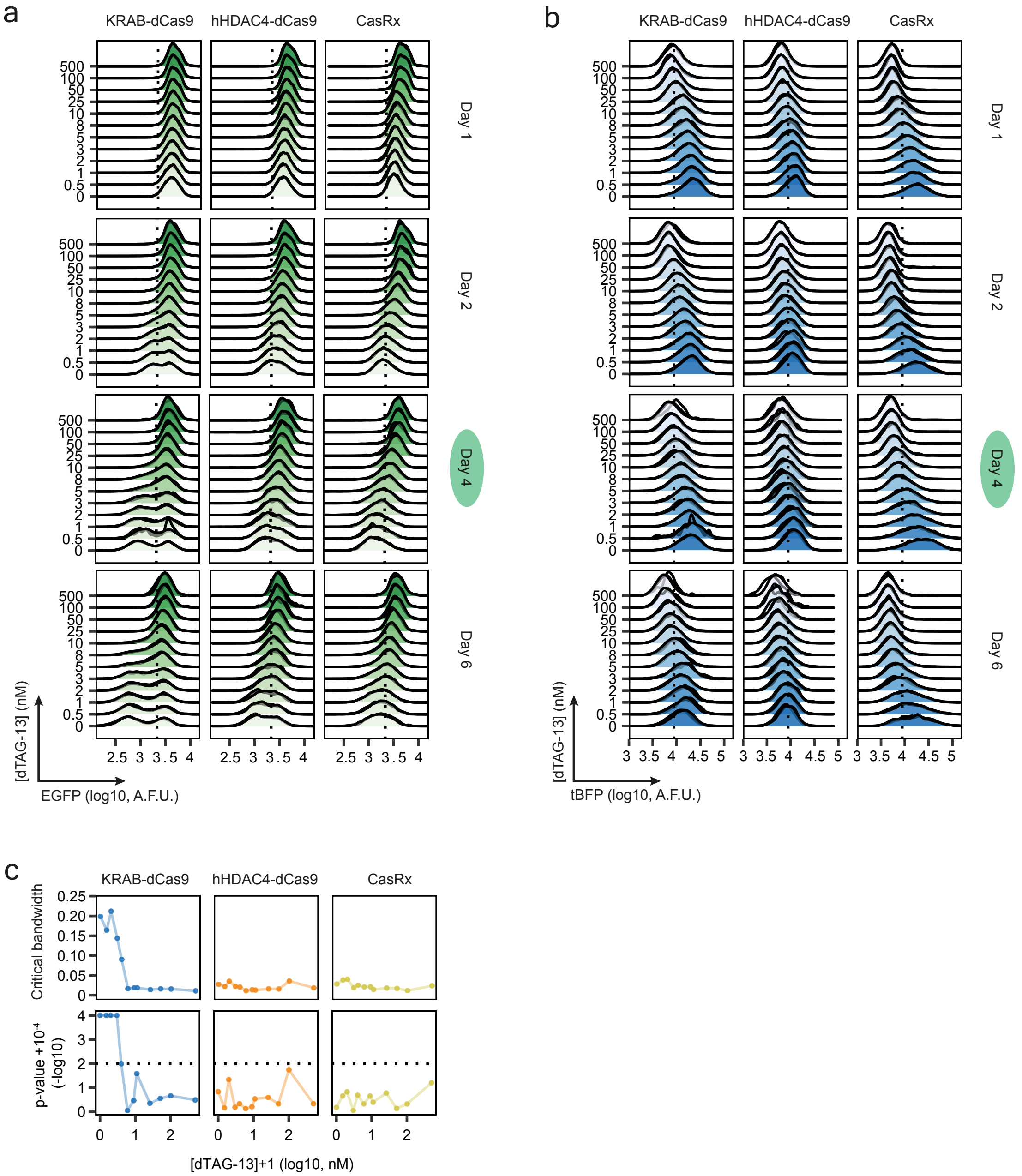

Supplementary Figure 5: Assessing tunability of degron-Cas-repressor systems

a, Density plots of STAG2-EGFP expression levels measured by flow cytometry at increasing dTAG-13 concentrations (y-axis, in nM) for degron-Cas-repressors indicated on top in cells expressing STAG2-targeting guides at 1 to 6 days of treatment, as indicated on the right. Day 4, indicated with a green oval, was used for all the comparisons, including the test in (**c**).

b, Same as in a but for tBFP (degron-Cas-repressors levels).

c, Test for unimodality (or lack thereof) of the EGFP distributions shown in (**a**). Critical bandwidth (top) and associated p-value (bottom) at different dTAG-13 concentrations, calculated with the Hall-York test for unimodality (see Methods for details). Null hypothesis is that distributions are unimodal. The horizontal dotted line indicates a p-value of 0.01.

Supplementary Figure 6

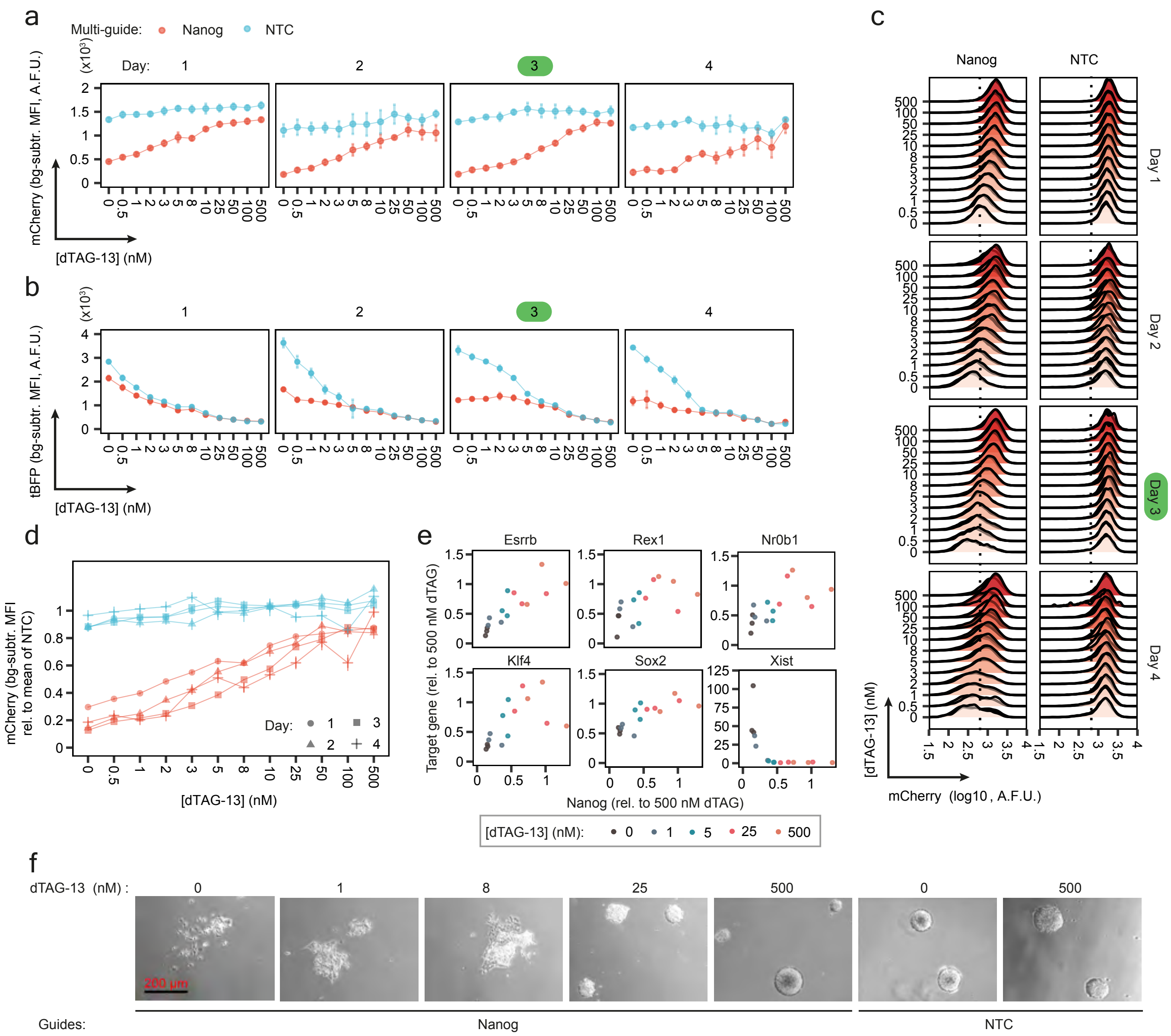

Supplementary Fig. 6: NANOG dose-response curves measured using CasTuner

a, **b**, Background-subtracted MFI for mCherry (**a**) or tBFP (**b**) in Nanog-mCherry mESCs expressing the hHDAC4-dCas9 CasTuner system and Nanog- (red) or non- (blue) targeting guides, at different dTAG-13 concentrations. The 4 panels from left to right represent measurement performed after 1 to 4 days from induction of knock-down. Dots show the mean of three biological replicates and vertical lines the standard deviation. Nanog knock-down results in reduced CasTuner expression, potentially because cells with strong Nanog knock-down cannot survive under ES cell conditions.

c, mCherry levels measured by flow cytometry in cells treated with the indicated dTAG-13 concentrations with Nanog-targeting guides (left) or NTC guides (right). Data from measurements at day 1 to 4 from knock-down

induction, as indicated on the right. In (**a**-**c**) the time point used for analysing Nanog dose-response curves is marked in green.

d, Nanog-mCherry expression in cells expressing targeting (red) or non-targeting guides (blue), at different dTAG-13 concentrations (x-axis) measured by flow cytometry. Symbols indicate different measurement time points as indicated. Symbols show the mean of three biological replicates and vertical lines the standard deviation.

e, Dose-response relationship for NANOG target genes at varying Nanog levels measured by qPCR. Individual measurements are shown as dots with colours indicating the applied dTAG-13 concentration.

f, Examples of colony morphologies in NANOG-titrated cells at different dTAG-13 concentrations, after 6 days of clonal growth. Scale bar= 200µm. The experiment is repeated 3 times independently with similar results.

Supplementary Figure 7

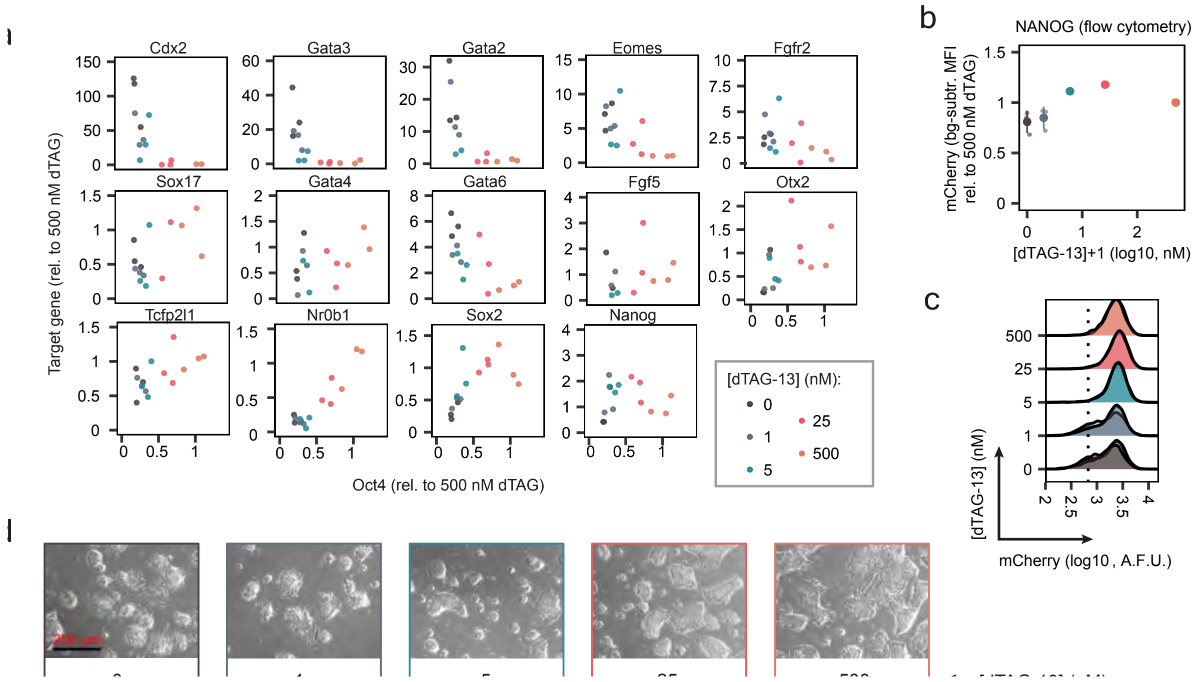

Supplementary Fig.7: Titrating Oct4 with CasTuner reveals a threshold level to induce the trophectoderm transcriptional program

a, Dose-response relationship for selected genes at varying Oct4 levels measured by qPCR. Individual measurements are shown as dots with colours indicating the applied dTAG-13 concentration.

b, NANOG expression quantified by flow cytometry after 3 days of Oct4 titration. Small dots show single replicates, bigger dots the mean of three biological replicates and vertical lines the standard deviation. Colours indicate the dTAG-13 concentration as in (**a**).

c, Density plots of NANOG-mCherry fluorescence at different dTAG-13 concentrations. Three biological replicates are overlaid.

d, Examples of cell morphologies at varying dTAG-13 concentrations after 3 days of titration OCT4 titration. Scale bar= 200µm. The experiment is repeated 3 times independently with similar results.
