# Supplementary material for: CasTuner is a degron and CRISPR/Cas-based toolkit for analog tuning of endogenous gene expression
Source: Nat Commun. 2023 Jun 3;14:3225. doi: 10.1038/s41467-023-38909-4 (PMC10239436; doi:10.1038/s41467-023-38909-4)
Supplement: Supplementary file 2 — Reporting Summary [file 41467_2023_38909_MOESM2_ESM.pdf]

nature portfolio

Corresponding author(s): Edda G. Schulz

Last updated by author(s): May 14, 2023

Reporting Summary

Nature Portfolio wishes to improve the reproducibility of the work that we publish. This form provides structure for consistency and transparency in reporting. For further information on Nature Portfolio policies, see our Editorial Policies and the Editorial Policy Checklist.

Statistics

For all statistical analyses, confirm that the following items are present in the figure legend, table legend, main text, or Methods section.

n/a	Confirmed	
☐	☒	The exact sample size (*n*) for each experimental group/condition, given as a discrete number and unit of measurement
☐	☒	A statement on whether measurements were taken from distinct samples or whether the same sample was measured repeatedly
☐	☒	The statistical test(s) used AND whether they are one- or two-sided<br>*Only common tests should be described solely by name; describe more complex techniques in the Methods section.*
☒	☐	A description of all covariates tested
☒	☐	A description of any assumptions or corrections, such as tests of normality and adjustment for multiple comparisons
☐	☒	A full description of the statistical parameters including central tendency (e.g. means) or other basic estimates (e.g. regression coefficient) AND variation (e.g. standard deviation) or associated estimates of uncertainty (e.g. confidence intervals)
☐	☒	For null hypothesis testing, the test statistic (e.g. *F*, *t*, *r*) with confidence intervals, effect sizes, degrees of freedom and *P* value noted<br>*Give P values as exact values whenever suitable.*
☒	☐	For Bayesian analysis, information on the choice of priors and Markov chain Monte Carlo settings
☒	☐	For hierarchical and complex designs, identification of the appropriate level for tests and full reporting of outcomes
☒	☐	Estimates of effect sizes (e.g. Cohen's *d*, Pearson's *r*), indicating how they were calculated

Our web collection on statistics for biologists contains articles on many of the points above.

Software and code

Policy information about availability of computer code

Data collection	Flow cytometry data were collected using the BDFACSDiva™ software v8.0.1.1.
Data analysis	All the data analysis was performed using the statistical computing language R version 3.6.3 using these packages: deSolve_1.30<br>nlstools_2.0-0<br>wesanderson_0.3.6<br>ggsci_2.9<br>ggridges_0.5.3<br>RColorBrewer_1.1-2<br>ggcyto_1.14.1<br>flowWorkspace_3.34.1<br>ncdfFlow_2.32.0<br>openCyto_1.24.0<br>flowCore_1.52.1<br>extrafont_0.17<br>nlme_3.1-152<br>minpack.lm_1.2-1<br>egg_0.4.5<br>gridExtra_2.3<br>dplyr_1.0.8

tidyr_1.1.3
ggplot2_3.3.3
tidyverse_1.3.1 .
Code is available at : https://doi.org/10.5281/zenodo.7928748

For manuscripts utilizing custom algorithms or software that are central to the research but not yet described in published literature, software must be made available to editors and reviewers. We strongly encourage code deposition in a community repository (e.g. GitHub). See the Nature Portfolio guidelines for submitting code & software for further information.

Data

Policy information about availability of data

All manuscripts must include a data availability statement. This statement should provide the following information, where applicable:

- Accession codes, unique identifiers, or web links for publicly available datasets
- A description of any restrictions on data availability
- For clinical datasets or third party data, please ensure that the statement adheres to our policy

All raw data generated in this study are available upon request. All processed data presented in this study are provided as a Source Data file. Plasmids are available through Addgene. A minimal dataset has been deposited at https://github.com/EddaSchulz/CasTuner/ , with DOI: https://doi.org/10.5281/zenodo.7928748 .

Note to the editor:
Because of the large amount and type (flow-cytometry) of raw data in this article, we do not envision a simple way to make them available to readers but they can be shared upon request.

Human research participants

Policy information about studies involving human research participants and Sex and Gender in Research.

Reporting on sex and gender	N/A
Population characteristics	N/A
Recruitment	N/A
Ethics oversight	N/A

Note that full information on the approval of the study protocol must also be provided in the manuscript.

Field-specific reporting

Please select the one below that is the best fit for your research. If you are not sure, read the appropriate sections before making your selection.

[x] Life sciences [ ] Behavioural & social sciences [ ] Ecological, evolutionary & environmental sciences

For a reference copy of the document with all sections, see nature.com/documents/nr-reporting-summary-flat.pdf

Life sciences study design

All studies must disclose on these points even when the disclosure is negative.

Sample size	All experiments were performed three times with independent replicates, in order to apply statistics whereas relevant. This is the standard in the field. For flow cytoemtry measurement, at least 10.000 events were recorded for HTS-based measurements and at least 30.000 when measuring from tubes.
Data exclusions	No data where excluded
Replication	All experiments were performed three times with independent replicates. All attempts at replication were successful.
Randomization	No randomization was included when designing the study. Randomization is not relevant in this study because we assumed that the type of measurements we performed (mainly flow cytometry) are independent from each other.
Blinding	Blinding was not relevant and rather not possible for this study.

Reporting for specific materials, systems and methods

We require information from authors about some types of materials, experimental systems and methods used in many studies. Here, indicate whether each material, system or method listed is relevant to your study. If you are not sure if a list item applies to your research, read the appropriate section before selecting a response.

Materials & experimental systems

n/a	Involved in the study
☒	☐ Antibodies
☐	☒ Eukaryotic cell lines
☒	☐ Palaeontology and archaeology
☒	☐ Animals and other organisms
☒	☐ Clinical data
☒	☐ Dual use research of concern

Methods

n/a	Involved in the study
☒	☐ ChIP-seq
☐	☒ Flow cytometry
☒	☐ MRI-based neuroimaging

Eukaryotic cell lines

Policy information about cell lines and Sex and Gender in Research

Cell line source(s)	Female mouse embryonic stem cells. The 1.8XX cell lines containing endogenous fluorescent reporters (Esrrb or Nanog) were generated in our lab as described in a previously published study (https://doi.org/10.1186/s13059-021-02321-2) based on 1.8XX mESC provided by Joost Gribnau (Erasmus UMC). The TX1072 cell line was also generate in a previous study in the group of Edith Heard (EMBL) (http://dx.doi.org/10.1016/j.stem.2013.11.022). The STAG2-EGFP HeLa Kyoto cell line was generated by the Jan-Michael Peters lab (https://doi.org/10.1038/s41586-018-0518-z). The HEK293T cell line was purchased (Sigma Aldrich).
Authentication	Cell lines were authenticated in the respective studies mentioned above. Esrrb-mCherry, Nanog-mCherry and STAG2-EGFP transgene expression was verified by flow-cytometry.
Mycoplasma contamination	All cell lines tested negative for mycoplasma
Commonly misidentified lines (See ICLAC register)	No commonly misidentified lines were used.

Flow Cytometry

Plots

Confirm that:

☒ The axis labels state the marker and fluorochrome used (e.g. CD4-FITC).

☒ The axis scales are clearly visible. Include numbers along axes only for bottom left plot of group (a 'group' is an analysis of identical markers).

☒ All plots are contour plots with outliers or pseudocolor plots.

☒ A numerical value for number of cells or percentage (with statistics) is provided.

Methodology

Sample preparation	Cell samples for flow cytometry were harvested by washing with PBS, dissociation with trypsin for 7 minutes at 37°C and resuspension in serum-containing medium (DMEM (Sigma), 15% ESC-grade FBS (Gibco), 0.1 mM β-mercaptoethanol). The cell suspension was centrifuged for 5 minutes at 500xg at 4°C, resuspended in flow cytometry buffer (PBS, 10% ESC-grade FBS (Gibco), 0.5mM EDTA) and kept on ice.
Instrument	The samples were measured using BD FACSCelesta Cell Analyzer (Beckton Dickinson, IC-Nr.: 68186, Serial-Nr.: R66034500035) with 2-Blue6-Violet4-561YG laser configuration, equipped with BD High Throughput Sampler (HTS). For Fluorescence Associated Cell Sorting (FACS), a BD FACSAria Fusion sorter (Beckton Dickinson, IC-Nr.:68198, Serial-Nr.:R658282830001) with a 2B-5YG-3R-2UV-6V lasers configuration was used.
Software	Data were collected using the BD FACSDiva software and analysed using the programming language R with the open-source packages FlowCore and OpenCyto
Cell population abundance	When sorting cells to generate new cell lines, during the first round of sorting tipically 3-5% of cells are selected for sorting and during the second round 10-20%. The percentage varies across cell lines but in the case of this study it was relevant to keep the gating strategy exactly the same in order to be able to effectively compare the properties of different cell lines.

Gating strategy

The cells were sorted based on their tBFP level (BV421 parameter on FACSDiva software). The strategy for sorting was selected in order to obtain a high number of cells, with an as uniform as possible level of expression, while maintaining high expression levels of the constructs, clearly distinguishable from the fluorescent background of the cells.

☒ Tick this box to confirm that a figure exemplifying the gating strategy is provided in the Supplementary Information.
