# Supplementary material for: CasTuner is a degron and CRISPR/Cas-based toolkit for analog tuning of endogenous gene expression
Source: Nat Commun. 2023 Jun 3;14:3225. doi: 10.1038/s41467-023-38909-4 (PMC10239436; doi:10.1038/s41467-023-38909-4)
Supplement: Supplementary file 4 — Description of Additional Supplementary Files [file 41467_2023_38909_MOESM4_ESM.pdf]

Title: Supplementary Data 1.
Description: Reagents
Part A: Sequences of sgRNA for dCas9.
Part B: Sequences of sgRNA for CasRx.
Part C: Oligonucleotides sequences for cloning of CasRx guides.
Part D: qPCR primers.
Part E: Plasmid overview

Title: Supplementary Data 2.
Description: Raw counts of Alkaline-Phosphatase stained cells upon Nanog titration.

Title: Supplementary Data 3.
Description: Strategy for double-sorting by FACS of cell lines with degron-Cas-repressor systems.

Title: Supplementary Data 4.
Description: Maps of plasmids used in this study.
