# Supplementary material for: CasTuner is a degron and CRISPR/Cas-based toolkit for analog tuning of endogenous gene expression
Source: Nat Commun. 2023 Jun 3;14:3225. doi: 10.1038/s41467-023-38909-4 (PMC10239436; doi:10.1038/s41467-023-38909-4)

Description: Strategy for double sorting of degron-Cas-Repressor systems
Cells are first sorted gating on BFP levels with a wider gate (BFP+), expanded and then sorted again (M population).
*Page 2: Example of first sorting. Sample: degron-dCas9-KRAB. Population sorted: BFP+
*Page 3: Example of second sorting. Sample: degron-dCas9-KRAB. Population sorted: M and Hi. Population M is used for all the experiments described in the manuscript.
The same gating coordinates are applied to all samples.

BD FACSDiva 8.0.1

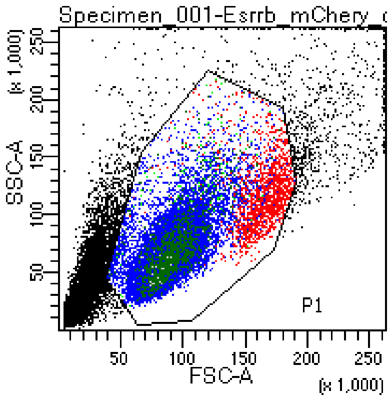

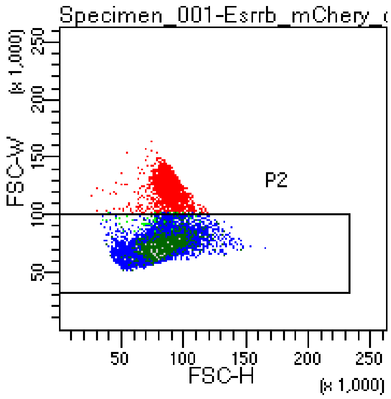

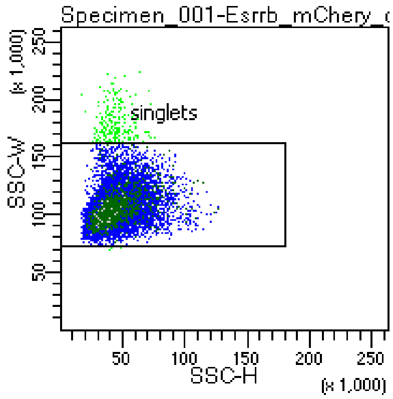

Tube: Esrrb_mChery_dCas9_dTAG_KRAB_BFP

Population	#Events	%Parent	%Total
All Events	37,981	####	100.0
P1	30,000	79.0	79.0
P2	28,317	94.4	74.6
singlets	28,078	99.2	73.9
BFP+	1,369	4.9	3.6
negative	27	0.1	0.1

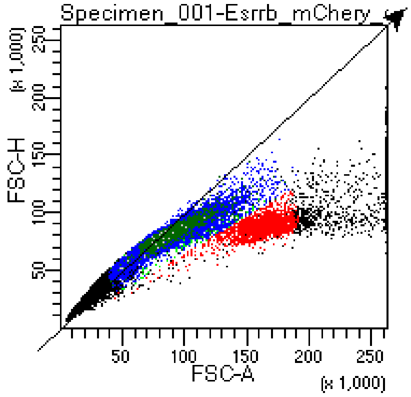

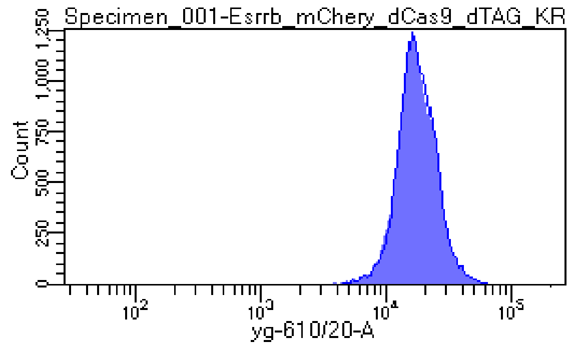

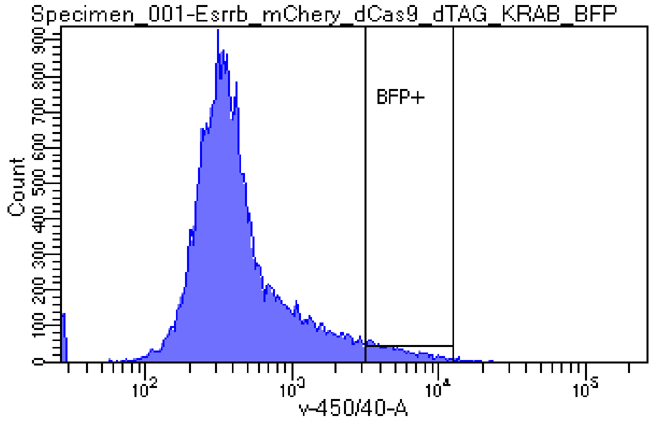

BD FACSDiva 8.0.1

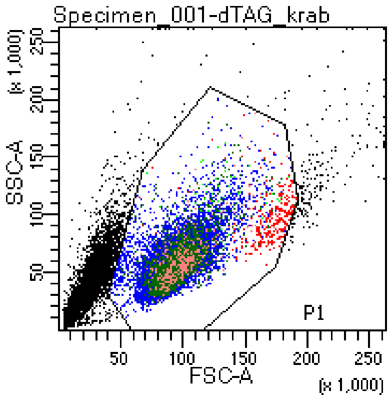

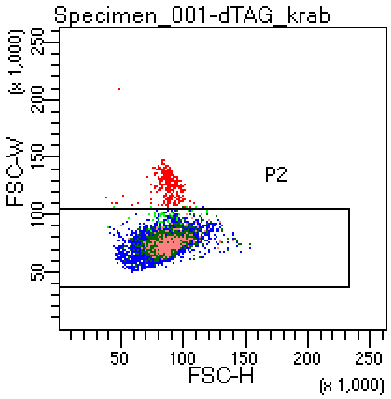

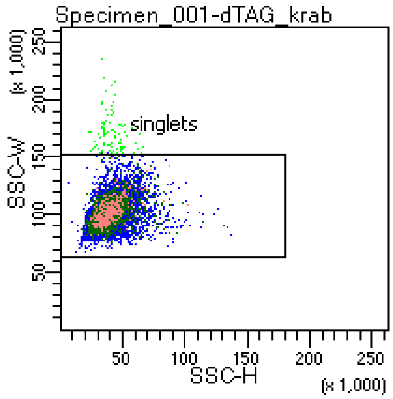

Tube: dTAG_krab

Population	#Events	%Parent	%Total
All Events	24,131	####	100.0
P1	20,000	82.9	82.9
P2	19,683	98.4	81.6
singlets	19,594	99.5	81.2
M	2,645	13.5	11.0
Hi	363	1.9	1.5

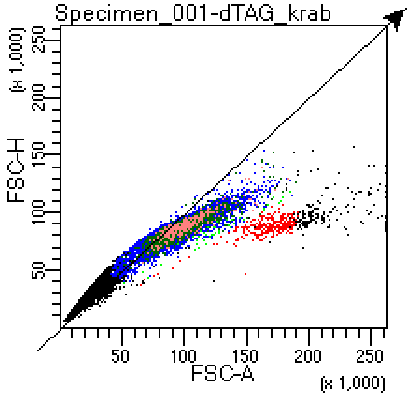

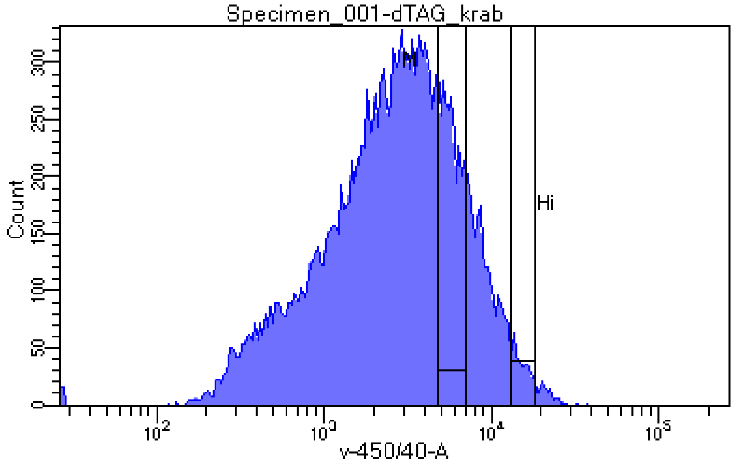

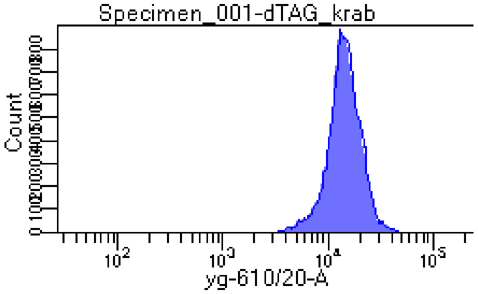

Supplement: Supplementary file 7 — Supplementary Data 3 [file 41467_2023_38909_MOESM7_ESM.pdf]
