# Supplementary material for: CasTuner is a degron and CRISPR/Cas-based toolkit for analog tuning of endogenous gene expression
Source: Nat Commun. 2023 Jun 3;14:3225. doi: 10.1038/s41467-023-38909-4 (PMC10239436; doi:10.1038/s41467-023-38909-4)

Short name:
dCas9-tagRFPt-P2A-tagBFP
Full name:
pSLPB2-SpdCas9-tagRFPt-P2A-tagBFP-PGK-Blasticidin

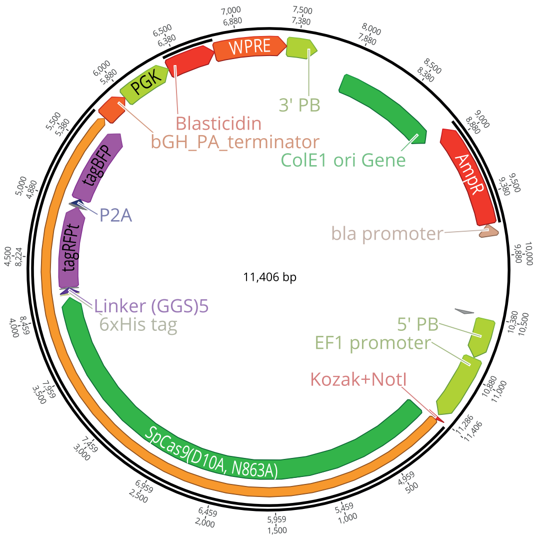

Short name:
AID-dCas9-tagRFPt-P2A-tagBFP
Full name:
pSLPB3B-AID-SpdCas9-tagRFPt-P2A-tagBFP-PGK-Blasticidin-T2A-osTIR1

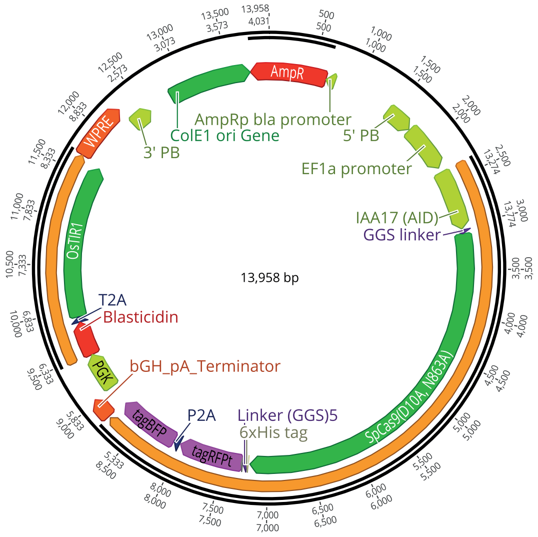

Short name:
mAID-dCas9-tagRFPt-P2A-tagBFP
Full name:
pSLPB3B-AID-SpdCas9-tagRFPt-P2A-tagBFP-PGK-Blasticidin-T2A-osTIR1

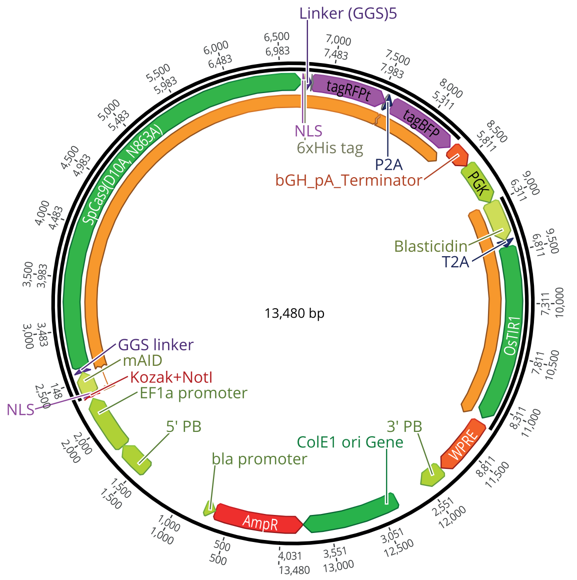

Short name:
ER50-dCas9-tagRFPt-P2A-tagBFP
Full name:
pSLPB2B-ER50-SpdCas9-tagRFPt-P2A-tagBFP-PGK-Blasticidin

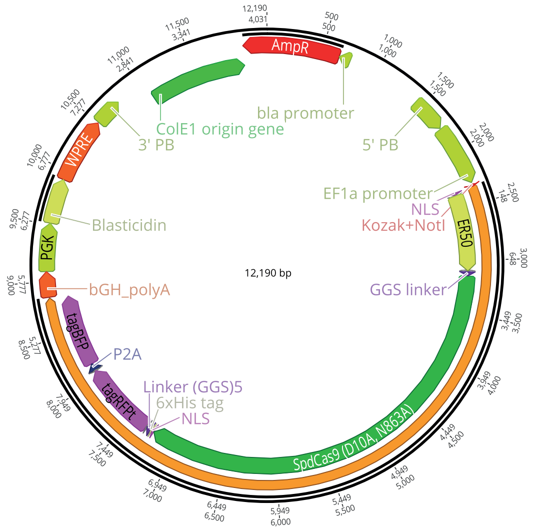

Short name:
SMASh-dCas9-tagRFPt-P2A-tagBFP
Full name:
pSLPB2B-SMASh-SpdCas9-tagRFPt-P2A-tagBFP-PGK-Blasticidin

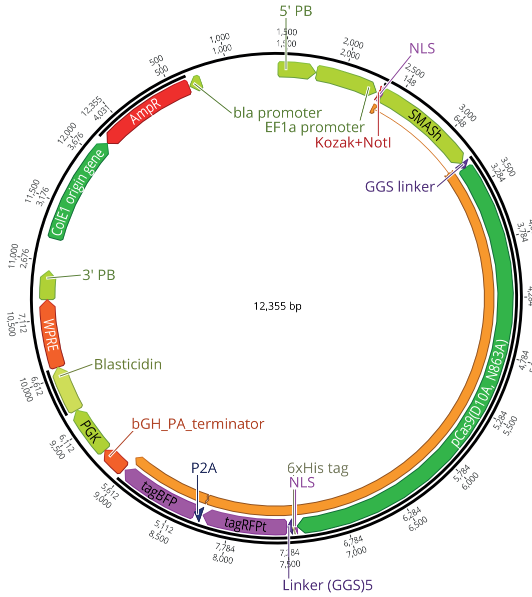

Short name:
ecDHFR-dCas9-tagRFPt-P2A-tagBFP
Full name:
pSLPB2B-ecDHFR-SpdCas9-tagRFPt-P2A-tagBFP-PGK-Blasticidin

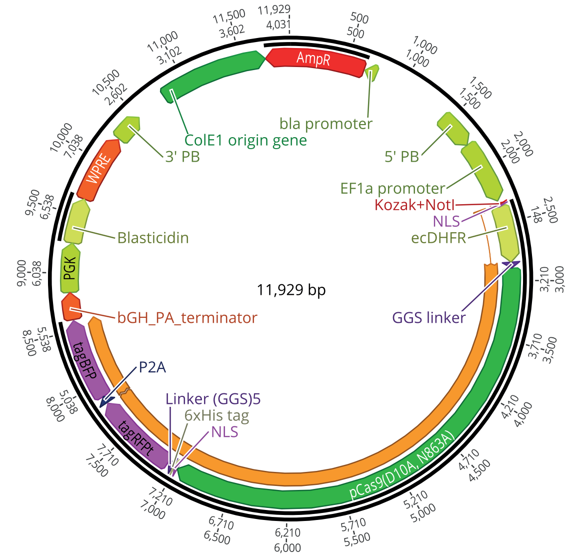

Short name:
FKBP12_F36V-dCas9-tagRFPt-P2A-tagBFP
Full name:
pSLPB2B-FKBP12_F36V-SpdCas9-tagRFPt-P2A-tagBFP-PGK-Blasticidin

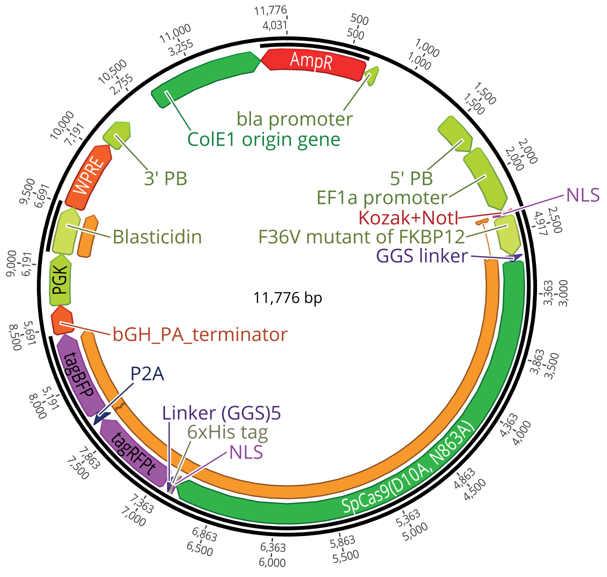

Short name:
FKBP12_F36V-CasRx-tagBFP
Full name:
pSLPB2B-FKBP12_F36V-CasRx-tagBFP-PGK-Blasticidin

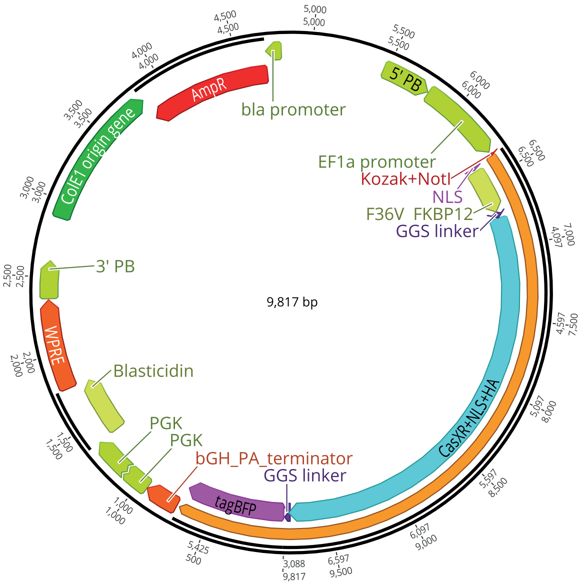

Short name:
FKBP12_F36V-dCas9-KRAB-tagBFP
Full name:
pSLPB2B-FKBP12_F36V-SpdCas9-KRAB-tagBFP-PGK-Blasticidin

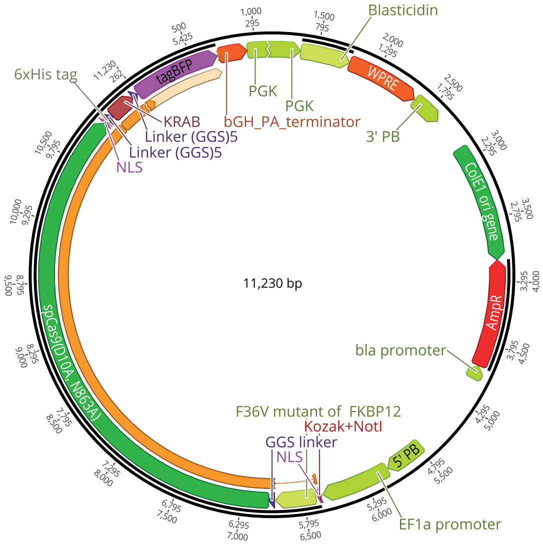

Short name:
FKBP12_F36V-dCas9-hHDAC4-tagBFP
Full name:
pSLPB2B-FKBP12_F36V-SpdCas9-hHDAC4-tagBFP-PGK-Blasticidin

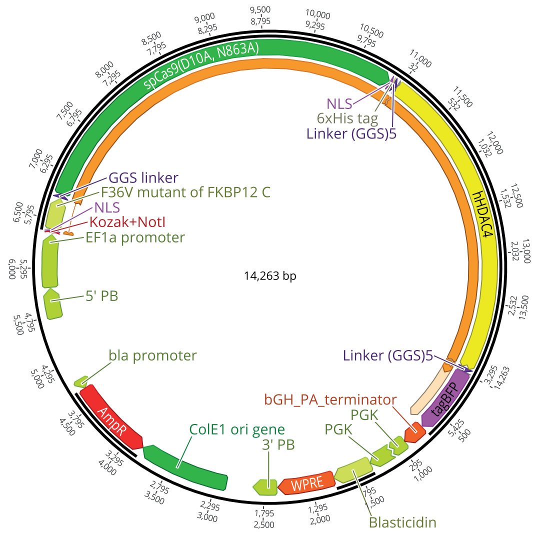

Short name:
FKBP12_F36V-KRAB-dCas9-tagBFP
Full name:
pSLPB2B-FKBP12_F36V-KRAB-SpdCas9-tagBFP-PGK-Blasticidin

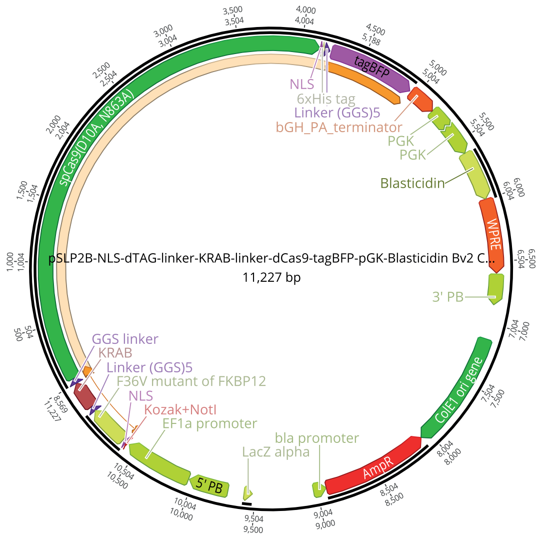

Short name:
FKBP12_F36V-hHDAC4-dCas9-tagBFP
Full name:
pSLPB2B-FKBP12_F36V-hHDAC4-SpdCas9-tagBFP-PGK-Blasticidin

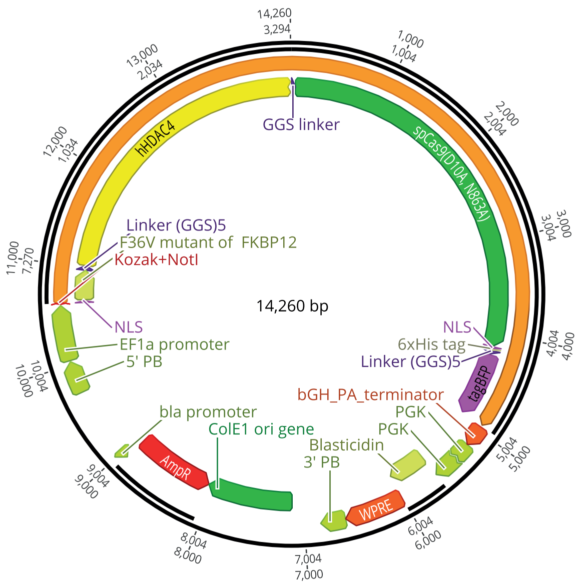

Short name:
KRAB-Split-dCas9-FKBP12_F36V
Full name:
pSLQ2818_Blasti_pPB: CAG-PYL1-KRAB-IRES-Blasti-WPRE-SV40PA
PGK-ABI-tagBFP-SpdCas9-FKBP_F36V

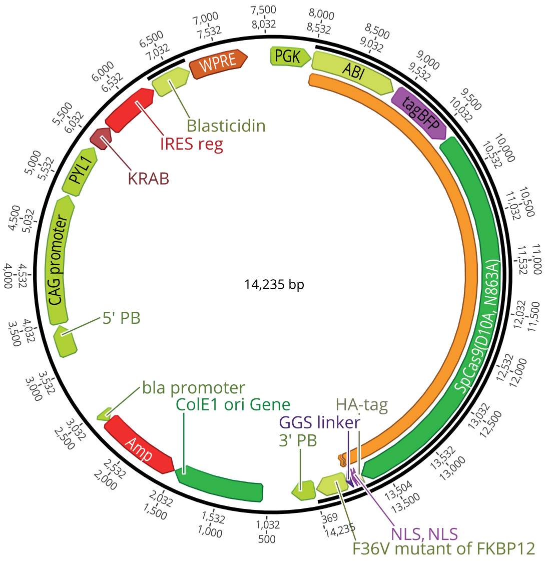

Supplement: Supplementary file 8 — Supplementary Data 4 [file 41467_2023_38909_MOESM8_ESM.pdf]
